# Comparison of Refraction Obtained with the Myopia Master and Subjective Manifest Refraction in Pediatric Orthokeratology

**DOI:** 10.3390/diagnostics16183022

**Published:** 2026-09-17

**Authors:** Cecilia Zamora-Castro, David P. Piñero Llorens, Elena Martínez Plaza

**Affiliations:** 1 Department of Optics, Pharmacology and Anatomy, University of Alicante, 03690 Alicante, Spain; cecilia.zamora@ua.es (C.Z.-C.); elena.martinez.plaza@uva.es (E.M.P.); 2 Department of Ophthalmology, Vithas Medimar International Hospital, 03016 Alicante, Spain; 3 Department of Theoretical Physics, Atomic and Optics, University of Valladolid, 47002 Valladolid, Spain; 4 IOBA (Institute of Applied Ophthalmobiology), University of Valladolid, 47002 Valladolid, Spain

**Keywords:** orthokeratology, Myopia Master, objective refraction, subjective refraction, myopia control, pediatric myopia, autorefractor

## Abstract

**Background:** Objective refraction plays an increasingly important role in paediatric myopia control. However, evidence regarding its agreement with subjective refraction in children undergoing orthokeratology (OK), particularly under non-cycloplegic conditions, remains limited. This study evaluated the longitudinal agreement between Myopia Master refraction obtained with the device and subjective manifest refraction during the first six months of OK treatment. **Methods:** A prospective longitudinal study included children aged 8–12 years fitted with OK lenses featuring a customized optical treatment zone. Subjective manifest refraction and Myopia Master refraction using the Myopia Master^®^ were performed at baseline and after 3 and 6 months under physiological pupillary conditions without cycloplegia. Agreement was assessed using paired comparisons, intraclass correlation coefficients (ICC), and Bland–Altman analysis. **Results:** Forty-three myopic participants were included at baseline. Excellent agreement was observed for sphere, spherical equivalent (M), and overall blur strength (B) (ICC ≥ 0.937), whereas cylinder and J_45_ showed lower agreement (ICC ≥ 0.701). Agreement decreased after OK treatment, particularly at 3 and 6 months. Bland–Altman analysis showed small mean bias with wider limits of agreement during follow-up. **Conclusions:** The Myopia Master^®^ showed clinically useful agreement for most subjects with subjective manifest refraction throughout the first six months of paediatric OK treatment. Despite reduced agreement after treatment initiation due to corneal remodelling, Myopia Master refraction remained a valuable complementary tool for routine follow-up under physiological pupillary conditions.

## 1. Introduction

Refractive errors are among the most common causes of visual impairment, with myopia being the most prevalent and rapidly increasing worldwide [1]. Current projections estimate that by 2050 nearly half of the global population will be myopic, accompanied by an increase in high myopia and the risk of associated pathological ocular changes [1,2]. Factors such as increased near work, reduced outdoor time and genetic predisposition contribute to the rapid rise in myopia prevalence, particularly in East Asian and European pediatric populations [3]. However, this growing prevalence has motivated the development of myopia-control strategies aimed at slowing myopia progression and reducing its long-term impact on pediatric ocular health. Interventions such as OK, multifocal soft contact lenses and pharmacologic treatments like low-dose atropine have demonstrated efficacy in slowing myopia progression [4].

OK is a reversible refractive correction technique based on the overnight wear of rigid gas-permeable contact lenses that induce temporary corneal reshaping, allowing functional unaided vision during daytime hours [5]. Beyond its refractive effect, numerous studies have demonstrated its efficacy in controlling myopia progression in pediatric populations, primarily through the slowing of axial length growth [6,7]. However, the corneal changes induced by OK, particularly in central and peripheral corneal curvature, pose significant challenges for refractive measurement and for the clinical interpretation of outcomes during treatment follow-up. Furthermore, given that the clinical response to OK constitutes a dynamic and progressive process requiring several days or weeks to achieve complete refractive stabilization [8], it is essential to have diagnostic methods capable of accurately monitoring these gradual changes at each follow-up visit.

To monitor these patients effectively, refractive errors are traditionally assessed by means of subjective manifest refraction, which is the clinical gold standard [9,10]. However, in recent years, several objective refraction platforms have become available, including conventional autorefractors, open-field systems, and wavefront-based aberrometers [9,10]. Myopia Master refraction provides several advantages over subjective assessment, such as reduced examiner dependence, greater standardization, and shorter testing times [11]. Current evidence suggests generally good agreement between objective and subjective refraction for most instruments, although differences may be more relevant in pediatric populations, particularly when measurements are obtained without cycloplegia [10,11]. However, differences between objective and subjective refraction have also been reported with wavefront-based devices, particularly when measurements are performed under cycloplegic conditions [12].

The Myopia Master^®^ (Oculus Optikgeräte GmbH, Wetzlar, Germany) is an advanced optical biometer that has gained relevance by integrating autorefraction, keratometry and axial length (AL) measurement into a single, rapid, non-contact device. Recent studies have shown that this system provides excellent repeatability for AL with variability around 0.02 mm [13], as well as high agreement with established biometers such as the IOLMaster [14]. In pediatric populations, a strong correlation has also been reported between cycloplegic measurements obtained with the Myopia Master, other biometric devices and manifest refraction, particularly for AL and keratometry (r > 0.9; ICC ≥ 0.943), as well as for spherical equivalent refraction (r > 0.920) [15]. However, evidence regarding the agreement of non-cycloplegic refractive measurements obtained with the Myopia Master in eyes undergoing corneal changes induced by OK remains limited, and most existing literature has focused on cross-sectional comparisons without longitudinal clinical follow-up.

In the clinical management of childhood myopia control with OK lenses, omitting routine cycloplegia during follow-up visits constitutes a fundamental strategic decision. Given that corneal reshaping and the clinical response to OK are gradual processes requiring several days or weeks to achieve complete refractive stabilization, pediatric patients must undergo multiple protocolized short- and medium-term control visits [5,16]. In this high-frequency follow-up setting, the repeated instillation of cycloplegic agents at each consultation generates a considerable iatrogenic burden, causing immediate stinging, discomfort, and unnecessary psychological and behavioural distress in children, while also disrupting their school performance and daily activities due to prolonged photophobia [17]. More importantly, from an optical perspective, pharmacological mydriasis expands the pupillary diameter beyond the limits of the central treatment optical zone moulded by the OK lens. When the pupil diameter exceeds this lens treatment zone, a massive increase in higher-order aberrations is induced, artificially degrading image quality and altering the habitual visual conditions under which the child utilizes OK-corrected vision, severely compromising the reliability and repeatability of clinical refraction [16,18].

Therefore, the primary aim of this study was to evaluate the longitudinal agreement between Myopia Master refraction measured with the Myopia Master device under physiological pupillary conditions and clinical subjective manifest refraction at baseline and after 3 and 6 months of OK lens wear in a pediatric population. The secondary objective was to assess the consistency and longitudinal stability of Myopia Master refractive measurements throughout the gradual corneal changes induced by OK treatment during clinical follow-up.

## 2. Materials and Methods

A prospective longitudinal study was conducted to evaluate the agreement between clinical subjective refraction and Myopia Master refraction obtained with the Myopia Master^®^ optical biometer (Oculus Optikgeräte GmbH, Wetzlar, Germany) in a pediatric myopic population before and after the initiation of OK treatment. The study was conducted as part of a clinical myopia control protocol with scheduled follow-up visits and adhered to the tenets of the Declaration of Helsinki. The study protocol was approved by the Research Ethics Committee “Comité Ético de Investigación con Medicamentos del Departamento de Salud Alicante–Hospital General” (Reference ISABIAL 2023-0363), and written informed consent was obtained from the parents or legal guardians of all participants after they received detailed information regarding the study procedures.

### 2.1. Participants

Participants were recruited from the Ophthalmology Department of Vithas Medimar Hospital (Alicante, Spain) between January and November 2025, as well as from participants responding to the recruitment call. Inclusion criteria comprised an age between 8 and 12 years, the presence of mild-to-moderate myopia, and a best-corrected visual acuity (BCVA) of 0.8 decimal or higher in both eyes. Exclusion criteria included a history of ophthalmic interventions or ocular surgery, active ocular diseases, systemic conditions affecting the ocular surface, any clinical contraindication to contact lens wear, and inadequate cooperation during the examinations.

### 2.2. Examination Protocol and Study Visits

A total of 45 participants were fitted with SeeFree^®^ (Conóptica-Hecht Contactlinsen, Barcelona, Spain) OK lenses with a customized optical treatment zone for overnight wear. Of these, two participants were excluded from the study analysis because the baseline examination revealed hyperopia and, therefore, they did not meet the myopia inclusion criterion. The remaining 43 participants met the eligibility criteria and were included in the baseline analysis. The present study specifically evaluated data collected at baseline, 3 months, and 6 months of lens wear. All examinations were performed by a single experienced optometrist following the same standardized clinical protocol throughout all study visits. At each of these visits, both clinical subjective manifest refraction and Myopia Master refraction with the Myopia Master biometer (Carl Zeiss Meditec, Jena, Germany) were performed. All measurements were obtained under physiological pupillary conditions without cycloplegia, thereby ensuring that the patient’s natural pupillary diameter was maintained, reflecting routine clinical practice.

### 2.3. Subjective Refraction

Subjective refraction was performed using a phoropter and trial lenses following a conventional clinical protocol, which included monocular refraction, binocular balance, and final refinement to achieve BCVA. BCVA was assessed using a standardized ETDRS chart with Sloan letters, with outcomes expressed in LogMAR units. All measurements were obtained without cycloplegia in order to reflect habitual manifest refraction in cooperative pediatric patients. Refractive outcomes were recorded as sphere, cylinder and axis. Subsequently, spherical equivalent, power vector components (M, J_0_ and J_45_) [19] and overall blur strength (B) [20] were calculated to allow quantitative comparison with the Myopia Master refraction data.

### 2.4. Myopia Master Refraction and Ocular Biometry

Myopia Master refraction and AL were obtained using the Myopia Master^®^, a system that integrates autorefraction and optical biometry based on low-coherence interferometry. Corneal curvature and topography parameters were measured using the Sirius corneal topographer (CSO, Florence, Italy). Both instruments allowed for a comprehensive, non-invasive assessment of the refractive and anatomical status of the eye. All measurements were obtained according to the manufacturers’ recommended acquisition protocols and subsequently used for statistical analysis.

### 2.5. Data Analysis

Descriptive statistics, expressed as mean standard deviation or median and interquartile range as appropriate, were used for sample characterization at baseline, 3 months and 6 months. To prevent statistical bias arising from inter-eye correlation, only one eye per participant was randomly selected for analysis. The normality of data distribution for the M, J0, J45 and the overall B across all three visits was evaluated using the Shapiro–Wilk test, paying particular attention to the 6-month visit to assess outcome stabilization.

To compare Myopia Master and subjective refractions at each follow-up visit, mean differences between the two methods were analyzed using the paired t-test for parametric data or the Wilcoxon signed-rank test for non-parametric datasets. A specific *p*-value was obtained for each individual comparison to detect statistically significant differences between the refraction methods. To evaluate clinical agreement, the Intraclass Correlation Coefficient (ICC) and Bland–Altman analysis were performed. ICC values were interpreted as poor (<0.50), moderate (0.50–0.75), good (0.75–0.90), or excellent (>0.90) [21]. The mean bias and the 95% limits of agreement (LoA) were calculated for baseline, 3-month, and 6-month data to determine the clinical discrepancy in diopters between the methods. For all statistical tests, a *p*-value of less than 0.05 was considered statistically significant. All statistical analyses were performed using SPSS software (version 26.0; IBM Corp., Armonk, NY, USA). A generative artificial intelligence tool (ChatGPT-5.6 Luna, OpenAI, San Francisco, CA, USA) was used solely for linguistic revision of the manuscript.

## 3. Results

### 3.1. Study Population Characteristics

A total of 43 eyes from 43 participants were included in the baseline analysis. The baseline demographic and clinical characteristics of the study population are summarized in Table 1.

### 3.2. Comparison Between Subjective and Myopia Master Refraction

The comparison between both methods showed differences depending on the follow-up visit and the refractive parameter evaluated (Table 2). At baseline, no statistically significant differences were found between subjective and Myopia Master refraction for sphere (*p* = 0.081), M (*p* = 0.510), the J_0_ component (*p* = 0.100), or B (*p* = 0.367). In contrast, statistically significant differences were observed for cylinder and the J_45_ component (both *p* < 0.001). At the 3-month follow-up, no statistically significant differences were found for J_0_ component (*p* = 0.251) or J_45_ component (*p* = 0.107). However, statistically significant differences were observed for sphere (*p* = 0.004), cylinder (*p* < 0.001), M (*p* < 0.001), and B (*p* < 0.001). At the 6-month follow-up, statistically significant differences were found for all parameters between both methods: sphere (*p* = 0.018), cylinder (*p* < 0.001), M (*p* < 0.001), J_0_ (*p* = 0.047) and J_45_ (*p* < 0.001) components, and B (*p* < 0.001).

### 3.3. Agreement Between Subjective and Myopia Master Refraction

The agreement between subjective and Myopia Master refraction was assessed using the ICC, and the results are summarized in Table 3. At baseline, excellent agreement was observed for sphere, M, and B, whereas good agreement was found for J_0_ component. In contrast, cylinder and J_45_ component showed moderate agreement. At the 3-month follow-up, the level of agreement decreased compared with baseline. Good agreement was observed only for cylinder, whereas sphere, M, J_45_ component, and B showed moderate agreement. The J_0_ component demonstrated poor agreement. At the 6-month follow-up, moderate agreement was observed for sphere, cylinder, M, J_45_, and B, while J_0_ component showed poor agreement.

### 3.4. Bland–Altman Analysis

**Figure 1:**
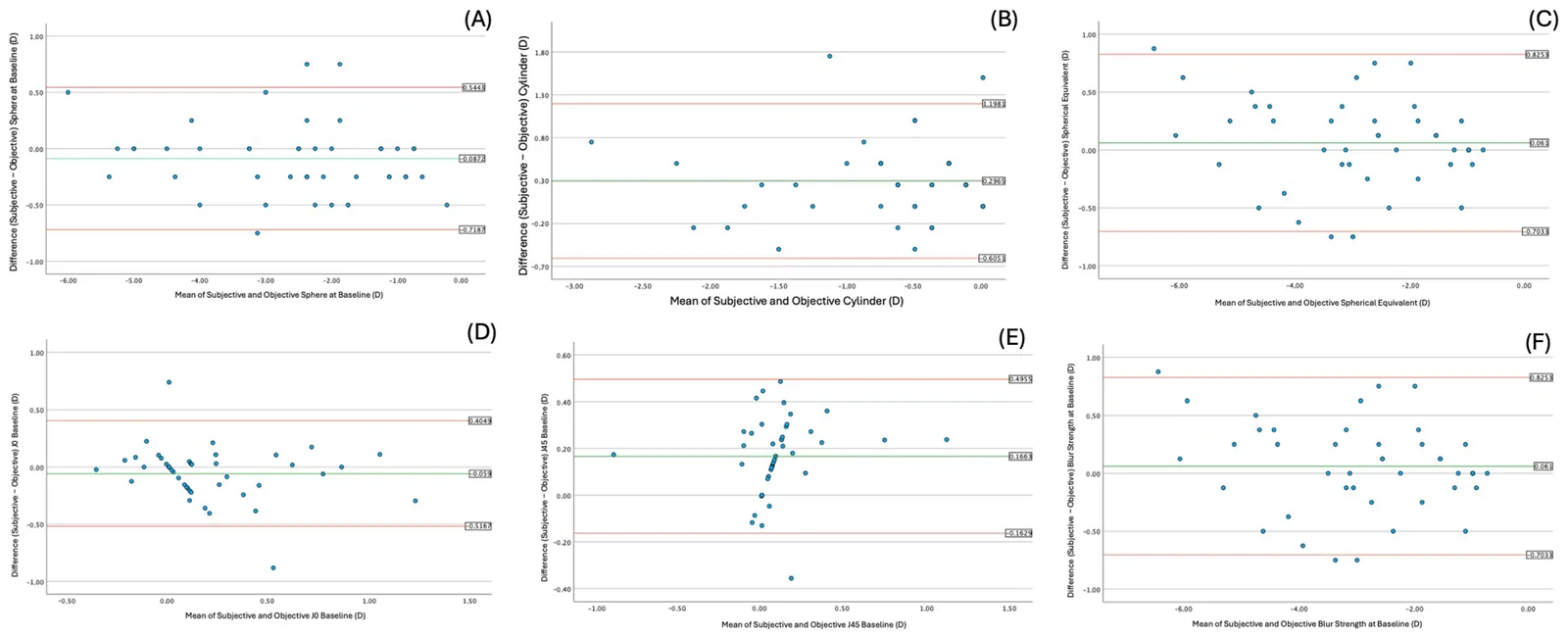
Bland–Altman plots comparing subjective and Myopia Master refraction at baseline. (**A**) Sphere. (**B**) Cylinder. (**C**) Spherical equivalent (M). (**D**) J_0_ component. (**E**) J_45_ component. (**F**) Blur strength (B).

**Figure 2:**
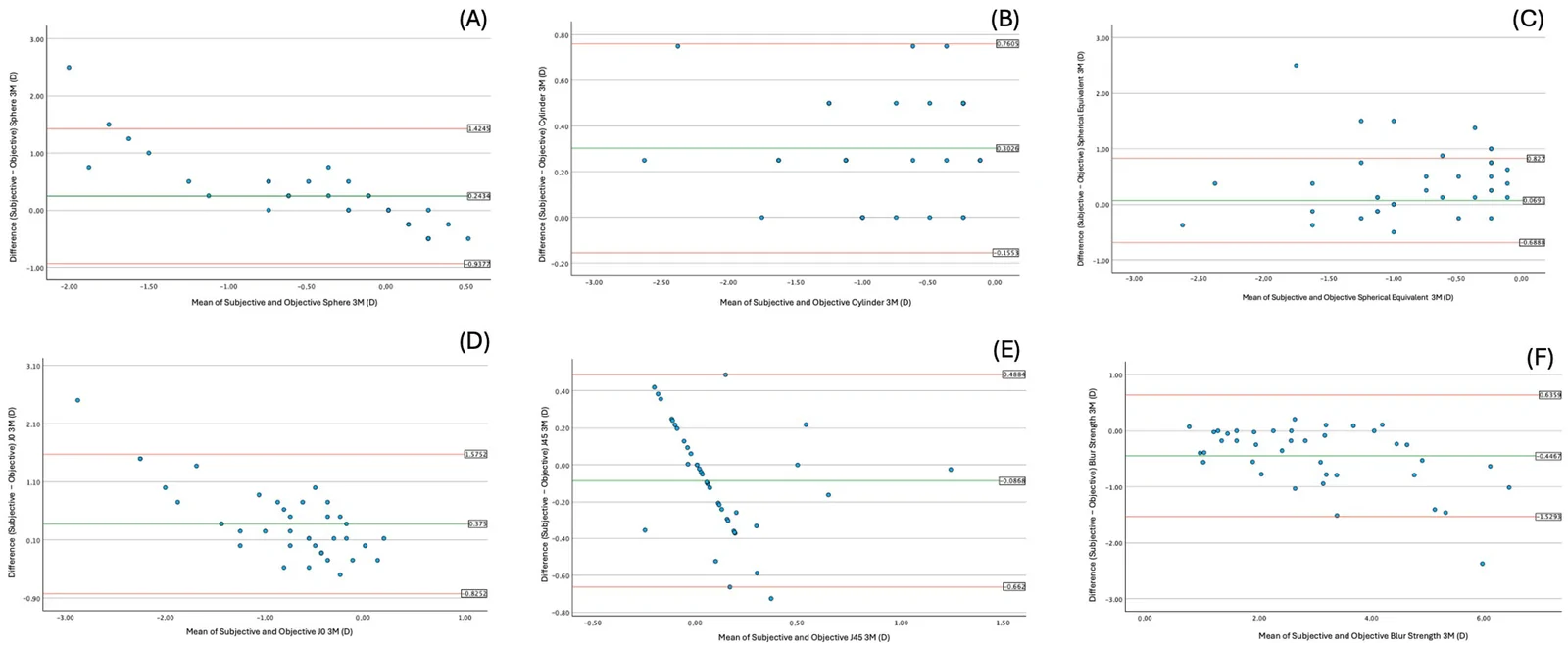
Bland–Altman plots comparing subjective and Myopia Master refraction at 3 months. (**A**) Sphere. (**B**) Cylinder. (**C**) Spherical equivalent (M). (**D**) J_0_ component. (**E**) J_45_ component. (**F**) Blur strength (B).

**Figure 3:**
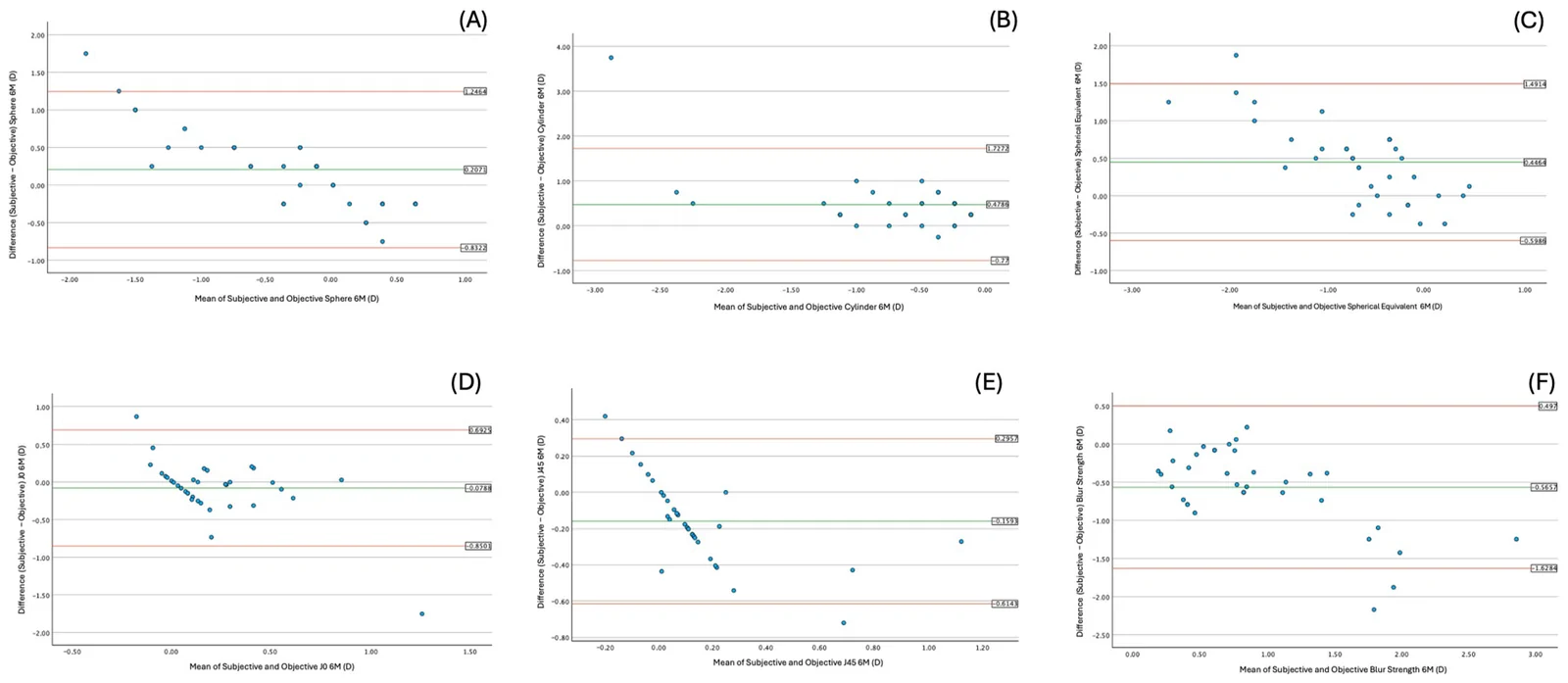
Bland–Altman plots comparing subjective and Myopia Master refraction at 6 months. (**A**) Sphere. (**B**) Cylinder. (**C**) Spherical equivalent (M). (**D**) J_0_ component. (**E**) J_45_ component. (**F**) Blur strength (B).

Bland–Altman analysis was performed to assess the agreement between subjective and Myopia Master refraction at baseline and at the 3- and 6-month follow-up visits. The corresponding Bland–Altman plots are presented in Figure 1, Figure 2 and Figure 3, and the mean bias and limits of agreement are summarized in Table 4. At baseline, most refractive parameters showed a mean bias close to zero, with relatively narrow limits of agreement, indicating good agreement between both measurement methods. At the 3-month follow-up, the mean bias increased for some parameters and was accompanied by wider limits of agreement, particularly for sphere, M, and B. At the 6-month follow-up, a similar pattern was observed, with a slight increase in the mean bias and wider limits of agreement compared with baseline, reflecting lower agreement between both methods.

## 4. Discussion

The present study evaluated, for the first time, the longitudinal agreement between subjective manifest refraction and Myopia Master refraction obtained with the Myopia Master under physiological pupillary conditions in children undergoing OK treatment. The results demonstrated good overall agreement between both methods throughout the follow-up period, although the level of agreement decreased after the initiation of OK for some refractive parameters. To evaluate the level of agreement, the classification scheme proposed by Koo and Li [21] was adopted. Under this scheme, the degree of agreement is categorized based on the 95% confidence interval of the ICC estimate as follows: poor for values below 0.5, moderate for values ranging from 0.5 to 0.75, good for values between 0.75 and 0.9, and excellent for values exceeding 0.90. In our sample, at baseline, all ICCs were over the value of 0.7. At 3 months of OK treatment, all ICCS were over 0.50 except for J_0_. This confirms some level of variability in the estimation of astigmatism, but not in the sphere. Despite the differences between refraction procedures, the Myopia Master refraction remained clinically acceptable (within a range of ±0.75 D) for most cases when compared with subjective refraction, supporting the potential utility of the Myopia Master as a complementary tool for the routine clinical follow-up of paediatric patients undergoing OK treatment. Specifically, eyes with greater residual myopia during OK treatment showed the largest discrepancies between refraction methods. These were the eyes with the highest level of myopia correction and, consequently, greater induction of spherical aberration. This can lead to errors in the estimation of the level of defocus using Zernike polynomials, as occurs with aberrometers and other objective refraction devices [22].

The slight reduction in agreement observed during follow-up was expected and can be explained by the progressive corneal remodelling induced by OK, as previously commented. Progressive corneal remodelling modifies the distribution of refractive power and corneal geometry, generating optical changes that may influence the accuracy of Myopia Master refraction and the agreement between both refraction methods [6,7]. Furthermore, the increase in higher-order aberrations, especially the primary spherical aberration, and the redistribution of the corneal profile reported following OK treatment may contribute to these discrepancies, particularly once the refractive effect of the lenses has been established [8,16]. Nevertheless, despite these changes, the findings of the present study indicate that the Myopia Master can maintain a clinically useful level of agreement throughout the follow-up period, but some care should be taken in eyes in which the induction of aberrations is higher (higher intended refractive correction). Guo et al. [23] reported that orthokeratology induces significant changes in corneal and ocular optics, including increased higher-order aberrations and spherical aberration, with the magnitude of these changes being influenced by the treatment zone size. These optical changes may contribute to differences between objective and subjective refraction after orthokeratology, as observed in our series.

The reliability of the device does not seem to be a critical factor underlying the differences between refraction procedures observed in the current study. Previous reports have demonstrated the high accuracy and reproducibility of the Myopia Master for the assessment of refractive and biometric parameters in paediatric populations. Martínez-Plaza et al. [13] reported excellent repeatability of measurements obtained with this device in healthy children, whereas Ye et al. [15] and McConnell et al. [14] confirmed a high level of agreement with established reference methods for refractive and ocular biometric measurements. However, these studies were conducted in eyes without corneal changes induced by myopia control treatments. In contrast, the present study extends the available evidence by providing the first longitudinal clinical evaluation of the Myopia Master in children undergoing OK, a clinical scenario characterized by progressive changes in corneal geometry and refractive status. It should be noted that monitoring of refraction, as well as of axial length, is crucial during OK treatment, and new Myopia Master methods are needed to provide longitudinal evaluation of these parameters, since cycloplegic refraction cannot be performed at every visit, as it is uncomfortable for children. Moreover, children undergoing OK are initially monitored frequently (even on a weekly basis), so a reliable estimation of refraction is needed.

From a clinical perspective, these findings suggest that the Myopia Master may represent a useful tool for the routine follow-up of children undergoing OK treatment, with special care in eyes with higher induction of aberrations. Although subjective refraction remains the reference standard for refractive assessment, the acceptable agreement for most patients observed between both methods supports the use of Myopia Master refraction as an adjunct during follow-up visits. This may be particularly relevant in patients undergoing long-term myopia control treatments, such as OK or low-dose atropine, in whom regular monitoring of refractive status is essential. In this context, obtaining reliable Myopia Master refraction under physiological conditions may optimize clinical follow-up by reducing the need for repeated cycloplegic examinations during routine visits, thereby minimizing the iatrogenic burden and patient discomfort associated with this procedure for both children and their families.

The present study has several strengths. To the best of our knowledge, it is the first to longitudinally evaluate the agreement between subjective refraction and refraction obtained with the Myopia Master in children undergoing OK treatment with a customized optical treatment zone, assessing this relationship from treatment initiation through the first six months of follow-up. Nevertheless, several limitations should be acknowledged. First, the follow-up period was limited to six months, precluding the assessment of the long-term evolution of agreement between both methods and the potential influence of longer-term refractive changes. Furthermore, the results were obtained from a single pediatric population treated with a specific OK lens design featuring a customized optical treatment zone; therefore, caution should be exercised when extrapolating these findings to other populations, lens designs, or devices. The potential influence of the customized optical treatment zone could be further evaluated by comparing these findings with a control group treated with conventional OK lens designs, using data available in our database. The potential use of short-acting mydriatics as an alternative strategy could also be evaluated in future studies. Future studies including longer follow-up periods, larger cohorts, and different myopia control strategies are warranted to confirm and extend these findings.

In conclusion, the findings of the present study demonstrate that the Myopia Master shows acceptable agreement for most patients with subjective refraction throughout the follow-up of children treated with OK lenses featuring a customized optical treatment zone, although this agreement decreases slightly after treatment initiation as a consequence of the optical changes induced by corneal remodelling. Nevertheless, the Myopia Master refraction obtained remains clinically useful as a complementary tool for the follow-up of these patients under physiological conditions.

## Figures and Tables

**Table 1:** Baseline characteristics of the study population.

Variable	Value
Age (years)	12.16 ± 1.99
Female, *n* (%)	24 (55.8)
Male, *n* (%)	19 (44.2)
Subjective sphere (D)	−2.63 ± 1.44
Subjective cylinder (D)	−0.58 ± 0.73
AL (mm)	24.44 ± 0.86
Corneal radius (mm)	7.83 ± 0.25

Data are presented as mean ± standard deviation (SD) for continuous variables and as *n* (%) for categorical variables.

**Table 2:** Comparison between subjective and Myopia Master refraction at baseline, 3 months, and 6 months.

Parameter	Baseline (*p*)	3 Months (*p*)	6 Months (*p*)
Sphere	0.081	0.004	0.018
Cylinder	<0.001	<0.001	<0.001
M	0.510	<0.001	<0.001
J_0_	0.100	0.353	0.047
J_45_	<0.001	0.069	<0.001
B	0.367	<0.001	<0.001

*p* values correspond to the comparison between subjective and Myopia Master refraction at each follow-up visit. Statistically significant differences were considered at *p* < 0.05.

**Table 3:** Intraclass correlation coefficients (ICC) between subjective and Myopia Master refraction at baseline, 3 months, and 6 months.

Parameter	Baseline ICC (95% CI)	3 Months ICC (95% CI)	6 Months ICC (95% CI)
Sphere	0.975 (0.953–0.986)	0.634 (0.385–0.795)	0.724 (0.505–0.853)
Cylinder	0.734 (0.420–0.870)	0.841 (0.097–0.952)	0.523 (0.108–0.758)
M	0.937 (0.886–0.965)	0.619 (0.286–0.800)	0.636 (0.153–0.838)
J_0_	0.781 (0.630–0.875)	0.395 (0.103–0.626)	0.362 (0.043–0.616)
J_45_	0.701 (0.121–0.881)	0.527 (0.267–0.717)	0.560 (0.173–0.775)
B	0.966 (0.939–0.982)	0.626 (0.170–0.826)	0.514 (−0.026–0.782)

Agreement was interpreted according to Koo and Li (2016) [21]: poor (<0.50), moderate (0.50–0.75), good (0.75–0.90), and excellent (>0.90).

**Table 4:** Mean bias and 95% limits of agreement between subjective and Myopia Master refraction at baseline, 3 months, and 6 months.

Parameter	Baseline Mean Bias (95% LoA)	3 Months Mean Bias (95% LoA)	6 Months Mean Bias (95% LoA)
Sphere	−0.087 (−0.719 to 0.544)	0.243 (−0.938 to 1.424)	0.207 (−0.832 to 1.246)
Cylinder	0.297 (−0.605 to 1.198)	0.303 (−0.155 to 0.761)	0.479 (−0.770 to 1.727)
M	0.061 (−0.703 to 0.825)	0.069 (−0.688 to 0.827)	0.446 (−0.599 to 1.491)
J_0_	−0.056 (−0.517 to 0.405)	0.375 (−0.825 to 1.575)	−0.079 (−0.850 to 0.693)
J_45_	0.166 (−0.163 to 0.496)	−0.087 (−0.662 to 0.488)	−0.159 (−0.614 to 0.296)
B	−0.078 (−0.849 to 0.692)	−0.447 (−1.529 to 0.636)	−0.566 (−1.628 to 0.497)

## Data Availability

The data presented in this study are available on request from the corresponding author due to privacy.

## Abbreviations

The following abbreviations are used in this manuscript:

AL	Axial Length
B	Blur Strength
D	Diopter
ICC	Intraclass Correlation Coefficient
J_0_	Jackson Cross-Cylinder at 0°/90°
J_45_	Jackson Cross-Cylinder at 45°/135°
M	Spherical Equivalent
OK	Orthokeratology
